# OptiMal-PK: an internet-based, user-friendly interface for the mathematical-based design of optimized anti-malarial treatment regimens

**DOI:** 10.1186/s12936-016-1401-8

**Published:** 2016-07-07

**Authors:** Ghaith Aljayyoussi, Katherine Kay, Stephen A. Ward, Giancarlo A. Biagini

**Affiliations:** Research Centre for Drugs and Diagnostics, Liverpool School of Tropical Medicine, Liverpool, L3 5QA UK; State University of New York at Buffalo, Buffalo, NY 14214 USA

**Keywords:** PK/PD modelling, Malaria, *Plasmodium*, Pharmacokinetics, Artemisinin, Drug discovery, Pre-clinical, Medicinal chemistry, Lead optimisation, ADMET, DMPK

## Abstract

**Background:**

The search for highly effective anti-malarial therapies has gathered pace and recent years have seen a number of promising single and combined therapies reach the late stages of development. A key drug development challenge is the need for early assessment of the clinical utility of new drug leads as it is often unclear for developers whether efforts should be focused on efficacy or metabolic stability/exposure or indeed whether the continuation of iterative QSAR (quantitative structure–activity and relationships) cycles of medicinal chemistry and biological testing will translate to improved clinical efficacy. Pharmacokinetic and pharmacodynamic (PK/PD)-based measurements available from in vitro studies can be used for such clinical predictions. However, these predictions often require bespoke mathematical PK/PD modelling expertise and are normally performed after candidate development and, therefore, not *during* the pre-clinical development phase when such decisions need to be made.

**Methods:**

An internet-based tool has been developed using STELLA^®^ software. The tool simulates multiple differential equations that describe anti-malarial PK/PD relationships where the user can easily input PK/PD parameters. The tool utilizes a simple stop-light system to indicate the efficacy of each combination of parameters. This tool, called OptiMal-PK, additionally allows for the investigation of the effect of drug combinations with known or custom compounds.

**Results:**

The results of simulations obtained from OptiMal-PK were compared to a previously published and validated mathematical model on which this tool is based. The tool has also been used to simulate the PK/PD relationship for a number of existing anti-malarial drugs in single or combined treatment. Simulations were predictive of the published clinical parasitological clearance activities for these existing therapies.

**Conclusions:**

OptiMal-PK is designed to be implemented by medicinal chemists and pharmacologists during the pre-clinical anti-malarial drug development phase to explore the impact of different PK/PD parameters upon the predicted clinical activity of any new compound. It can help investigators to identify which pharmacological features of a compound are most important to the clinical performance of a new chemical entity and how partner drugs could potentially improve the activity of existing therapies.

## Background

Anti-malarial drug development has entered a new era where the community can boast to be in possession of thousands of anti-malarial active compounds with in vitro growth inhibition IC_50_ values in the sub-micro molar range [1–3]. The increased activity in anti-malarial drug development within the context of the malaria eradication agenda, has resulted in the recommendation of a series of target candidate profiles (TCPs) and target product profiles (TPPs). The TCPs include; fast-parasite clearance drug profiles (TCP-1), long-duration of drug action profiles (TCP-2), liver-stage (including hypnozoites) and sexual-stage acting drug profiles (TCP-3), and chemoprotection profiles (TCP-4). The ambition is to combine drugs with one or more TCP attributes to either (i) generate a single exposure radical cure and prophylaxis (TPP-1, SERCaP) treatment- or (ii) generate a single exposure chemoprotection treatment (TPP-2, SECA) [4].

The challenge for the drug discovery community is to prioritize anti-malarial hits and leads for subsequent pre-clinical development. Lead optimization studies are used to enhance the effectiveness of the most promising compounds, however it is not always apparent whether a drug’s progress is directly in line with desired TCPs or TPPs. For example, despite the availability of in vitro ADME data (absorption, disposition, metabolism and excretion) or in vivo DMPK (pharmacokinetics) studies are unable to show whether a drug’s less desirable pharmacokinetic attributes can be counter-balanced by other more desirable features such as potency. Slow parasite killing kinetics (measured as the parasite reduction ratio, PRR) are clearly undesirable but can be overcome if the drug has an extensive therapeutic half-life. Similarly, a rapid-killing drug can be effective even with a short therapeutic half-life. Therefore to be confident of a drug’s ultimate clinical performance, extensive in vivo experimentation or pharmacokinetic–pharmacodynamic (PK/PD) modelling is required.

The major factors influencing the overall clinical performance of a drug include; (i) the drug’s therapeutic window describing the maximum dose tolerated by patients and the minimum dose required to produce a therapeutic effect, (ii) the drug’s PK properties describing its clearance and volume of distribution (ultimately reflected in the elimination half-life) and (iii) the drug’s PD properties, which include intrinsic potency (as determined in static growth inhibition assays) and time-dependent kill dynamics (in the case of malaria the PRR which is a fixed characteristic of the biological target/pathway affected by the anti-malarial drug class).

The complexity of interacting factors that affect the overall performance of a drug make it a challenge during lead optimization and pre-clinical drug development to fully understand when appropriate PK/PD optimization has been (or can ever be) achieved. Pharmacological models have the potential to bridge this knowledge gap and provide quantitative predictions of drug performance. In silico PK/PD models have historically been used to investigate anti-malarial monotherapies [5–10] and more recently combination therapies [11–14]. However, these studies do not offer a platform for non-specialists to explore the dynamic consequences of different pharmacological drug properties in order to support decision-making in the discovery process.

Presented here, is a user-friendly, internet-based platform “OptiMal-PK” that provides performance data that can support pre-clinical anti-malarial drug development initiatives. OptiMal-PK allows the user to input drug PK/PD parameters and utilizes a simple traffic-light display system to indicate the minimum number of days the drug should be administered to completely clear parasites form a patient. OptiMal-PK also allows for the optional addition of a partner drug from either a list of currently available drugs or a second user-defined drug. This allows the users to investigate how drug combinations could help shorten treatment times or alter regimens if needed. This tool serves two major purposes (i) to develop a better understanding of the complex interaction of anti-malarial drug PK and PD properties and (ii) to identify and quantify the key features of any drug under development that drive efficacy and therefore represent a focus for improvement in the drug development cycle.

The OptiMal-PK tool is an open access tool available online [15] which has been designed to be user friendly. Users can easily alter PK and PD values and generate instantaneous answers for any combination of PK/PD parameters with or without the addition of a partner drug. The model design, validation and examples of the uses of OptiMal-PK are described in the sections that follow.

## Methods

### Software

The software used for the construction of this model is STELLA^®^ (Systems Thinking for Education and Research) which is a package that allows for the dynamic visualization and communication of complex differential equation systems [16]. The model itself is hosted on forio.com, a platform for enabling simulations, data exploration and data analytics.

### The model

The model, based on Winter and Hastings [11] as previously described, connects a PK component describing the drug concentration in the systemic circulation over time and a PD component defining the effect of the drug concentration at any given time on the total parasite count. This allows the user to input the PK/PD parameters of their experimental drug and roughly estimate its predictive clinical performance in patients.

The PK model within OptiMal-PK assumes the drug is given orally, and because it is based on a previous model, it also assumes first-order absorption kinetics and distribution into one PK compartment. The model was hence only defined by two standard differential equations:

1 $$\frac{{dX_{1} }}{dt} = - k_{a} \cdot X_{1}$$

2 $$\frac{{dX_{2} }}{dt} = k_{a} \cdot X_{1} - k_{e} \cdot X_{2}$$

where *X*_1_ represents the mass of drug in the gut at time *t* and is at its maximum when a dose is administered. X_2_ is the mass of drug in the blood at any given time, it increases as the drug is absorbed from the gut at rate *k*_*a*_ and decreases as the drug is eliminated at a rate *k*_*e*_. Blood concentration levels are used throughout the model instead of plasma as the model assumes that drug elimination is driven by total clearance where protein binding does not alter overall exposure.

**Fig. 1:**
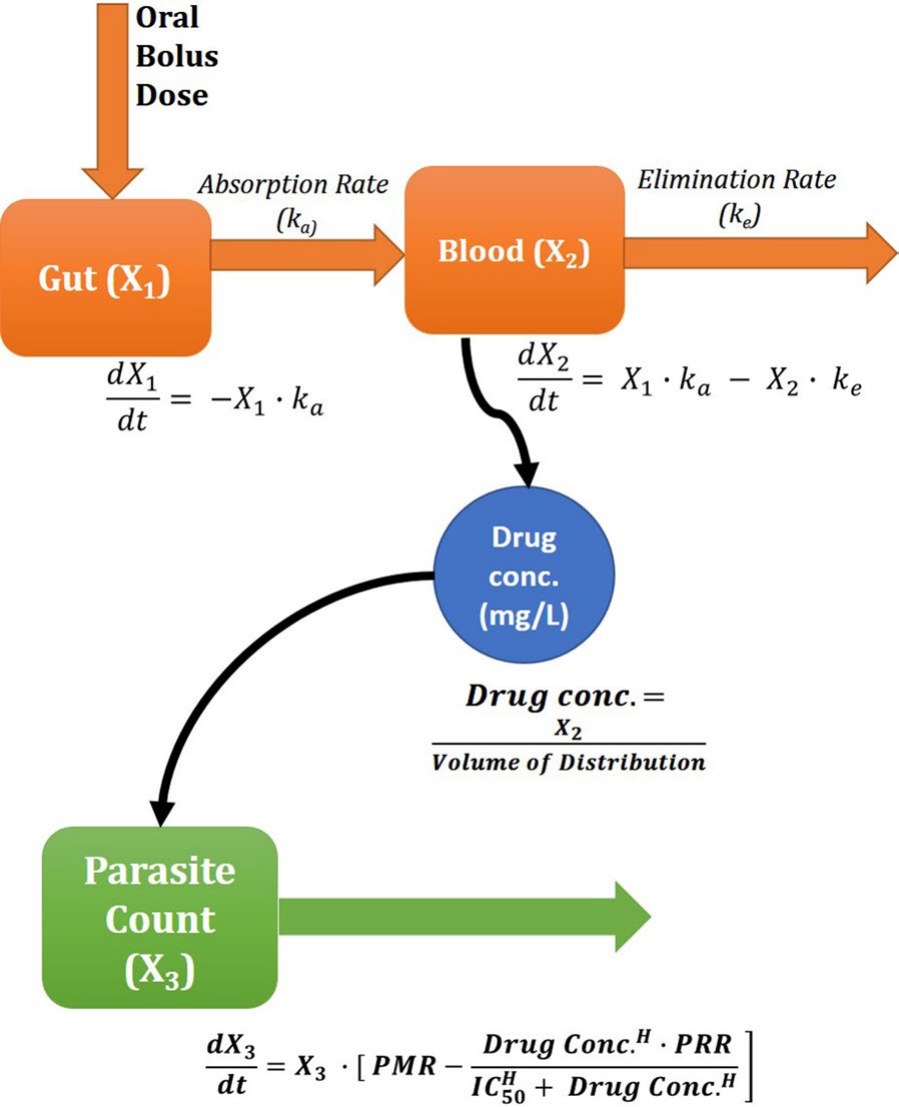
Outline of mathematical equations defining the OptiMal-PK model. Schematic visualising differential equations which define the pharmacokinetics of a drug where it is absorbed from X_1_ (Gut) to X_2_ (Blood) at a rate of *k*_*a*_ and eliminated at a rate of *k*_*e*_. Drug conc. is calculated at every time point as X_2_/volume of distribution. Parasite growth and drug induced death is defined by the dynamics of X_3_

Setting the dose in the interface will then change the value of X_1_ accordingly to match the required dosage. Initial value for X_2_ is set to be 0 by default as this represents initial drug amount in blood circulation as can be seen in Fig. 1.

The drug concentration (*C*) at any given time (*t*) is determined by dividing the drug mass in blood (*X*_*2*_) by the volume of distribution (*V*_*d*_).

3 $$C = \frac{{X_{2} }}{{V_{d} }}$$

This relationship can be simplified into terms for the apparent elimination half-life and the clearance of the drug; these parameters are required as user inputs are converted by the program into the micro-parameters as follows:

4 $$k_{e} = \frac{LN\left( 2 \right)}{{t_{1/2} }}$$

5 $$V_{d} = \frac{CL}{{k_{e} }}$$

The pharmacodynamic component of the model takes into account the parasite multiplication rate per parasite life cycle (PMR = parasite multiplication rate/48 h), the concentration of drug in blood at any specific time, the PRR (the maximum Parasite Reduction Ratio occurring in 48 h [11, 17–19]), C_50_ of the drug, defined as the concentration of the drug required to achieve half of the maximal parasite reduction rate and the Hill slope, which defines the slope parameter of the drug’s C_50_. The C_50_ value is the concentration required to achieve half the maximal PRR which is defined by the user. This value is ideally extracted from in vitro PRR studies [20], and if not available, the IC_50_ value derived from traditional static 48 or 72 h assays can be used as a suitable surrogate in OptiMal-PK [21]. Utilizing the IC_50_ values from static models have resulted in realistic predictions that match clinical results obtained from various drugs as previously shown [11].

The PD model relates drug concentration *C* to its effect on parasite viability. The concentration and time-dependent killing function *f*(*C*) for each drug is described using the standard Michaelis–Menten equation, i.e.

6 $$f\left( C \right) = V_{max} \left( {\frac{{C^{n} }}{{C^{n} + C_{50}^{n} }}} \right)$$

where *V*_*max*_ is the maximal drug-killing rate, *n* is the slope of the dose response curve, and *C*_50_ is the concentration of drug at which 50 % of the maximal PRR occurs.

7 $$V_{max} = - 0.5{\text{LN}}\left( {\frac{1}{PRR}} \right)$$

The change in the number of parasites *P* over time *t* can be found with the standard differential equation.

8 $$\frac{dP}{dt} = P\left( {a - f\left( C \right)} \right)$$

where (*a*) is the parasite growth rate determined by the user-defined parasite multiplication rate (PMR). PMR is set by default to ten based on previous evidence [22], but could be altered by the user to reflect the different PMR values that have been reported in different regions [23].

The model additionally calculates the minimum parasiticidal concentration (MPC), a term often used to describe the minimum concentration needed to achieve a net decrease in parasite count over time. MPC is directly calculated from the drug concentration (C) that results in a net reduction in parasite load (e.g. rate of parasite kill (*f*(*C*)) > PMR, Eq. 8).

9 $$a = - 0.5{\text{LN}}\left( {\frac{1}{PMR}} \right)$$

The model’s work-flow follows the schematic shown in Fig. 1.

Parameter values for all built in partner drugs supplied in the table (see OptiMal-PK website) were taken from the paper on which OptimMal-PK is based [11] except for atovaquone where the PK parameters were taken from [24], the IC_50_ data from [20] and the PRR values obtained from clinical data [25] which matches the in vivo PRR of drugs with similar mode of action [26].

Stage specificity within OptiMal-PK. A recent paper by Hodel et al. [27] investigated the accuracy of this methodology by modelling drugs with long and short half-lives, with and without stage specificity. The study found stage-specificity was only important for short half-life drugs with stage-specific killing (e.g. the artemisinins) because, depending on the timing of treatment, parasites might be in highly drug-tolerant stages or in much less tolerant stages. When modelling drugs with very short half-lives and stage-specific killing users should note that their results could vary but in all other instances, the model was shown to be very robust without the addition of stage-specificity.

OptiMal-PK has been designed to be a user-friendly tool for non-specialists allowing the potential activity of a developing drug or new drug combination to be assessed but care should be taken into over-interpretation of any results due to the previous complexities that have been necessarily simplified in the model.

### Interface

OptiMal-PK is an internet-based program that utilizes STELLA^®^ (Systems Thinking for Education and Research) software. The user-friendly interface allows scientists with limited knowledge of mathematical modelling and/or the PK-PD dynamics of anti-malarial drugs to easily assess the predicted clinical performance of any anti-malarial drug/drug combination and explore the consequence of modifying key pharmacological properties of the drug.

**Fig. 2:**
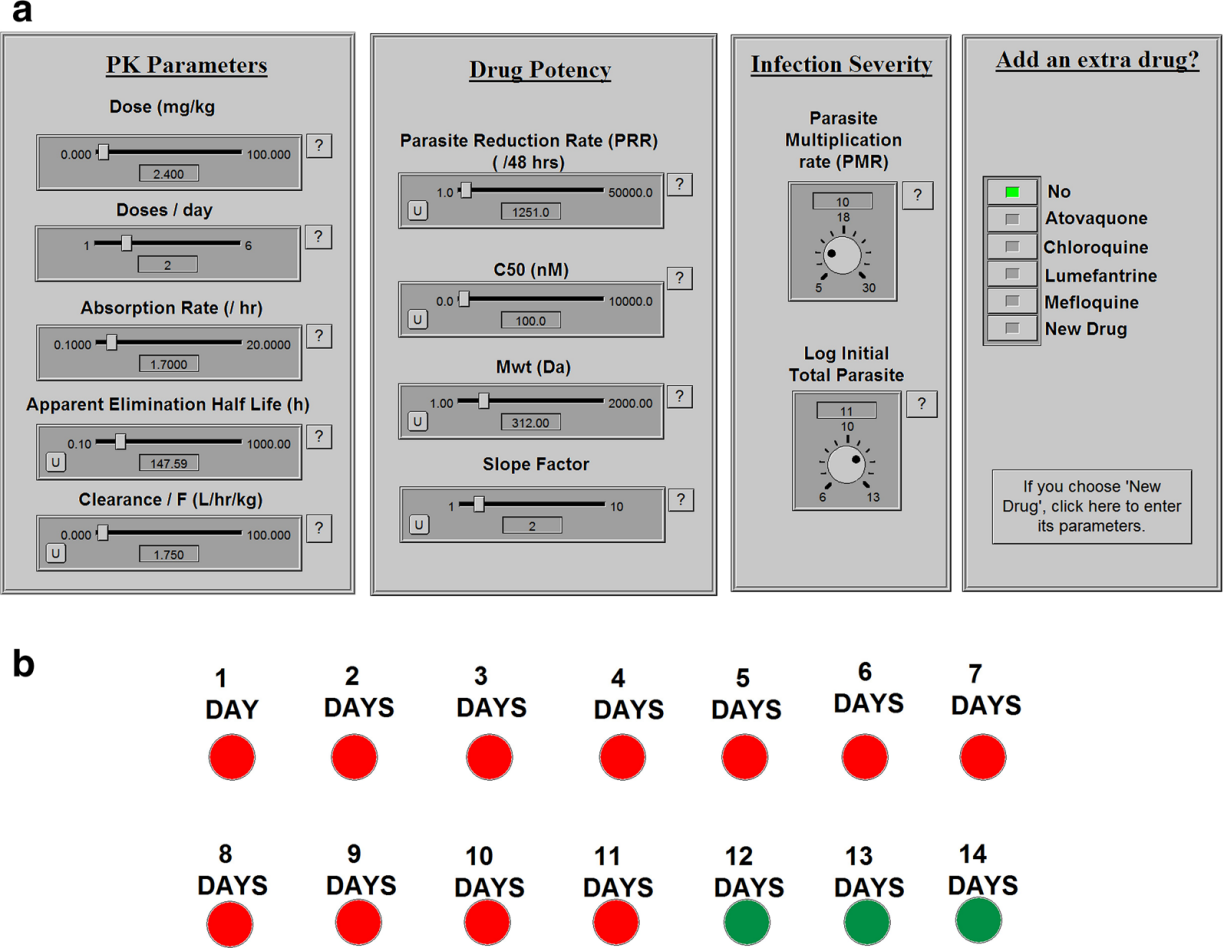
Interface of the OptiMal-PK tool. **a** Shows the panels where the user could use slide bars and turning knobs to adjust the PK parameters (dose, dosing frequency, absorption rate, apparent elimination half-life and clearance/F) and the PD parameters (Drug Potency) such as parasite reduction ratio (PRR), IC_50_, Mwt (to adjust for units of IC_50_ (moles) and dose (mgs) and the Slope factor (Hill’s Constant). Infection Severity Panel where the user could adjust the initial parasite count as well as the parasite multiplication ratio and finally a panel where the user could add an extra drug from a list of built-in drugs or a new drug whose parameters could be adjusted by the user. **b** Shows the output section of the tool, where upon running the simulation, the number of days of treatment required to clear parasites at the chosen PK/PD parameters will flash *green*, while the numbers of days that won’t be sufficient will flash *red*

The software works in two simple steps. First the user inputs the parameters describing drug PK, PD and initial parasite load with user-friendly dials, shown in Fig. 2a, and then runs the simulation by pressing the green “run” button (Fig. 2b). The OptiMal-PK simulation calculates the minimum treatment time (days) required to clear an infection and displays the results using a simple traffic light system. A green light indicates an adequate number of treatment days, i.e. the number of days of treatment is sufficient to achieve a cure (defined as reaching a total parasite count of less than one, where the simulation stops and indicates achievement of cure). While a red light indicates that the number of days of treatment administration is insufficient to achieve a cure.

## Results and discussion

### Model validation

The OptiMal-PK model was based on a validated mechanistic PK/PD model implemented in R [28] and previously published by Winter and Hastings [11] To validate the methodology of the OptiMal-PK model, the drug concentration and parasite number for the first 30 days post-treatment were simulated using both the OptiMal-PK and R models. The results of both simulations for the seven anti-malarial monotherapies previously calibrated and validated in [11] were compared in house and found to be largely consistent.

Small differences in the absolute values appear to be the result of the methods used by the modelling platforms in integrating differential equations. Stella^®^ uses the Euler’s method to solve differential equations in 1 h time steps while the Winter and Hastings model uses an algebraic solution for the differential equations. However, parasite levels at the selected time points were consistently within ±10 % across the two models.

The assumption of a one compartment model has been used previously to simulate three widely used anti-malarial drug combinations (artemether–lumefantrine, artesunate–mefloquine and DHA–piperaquine) [11]. However it is noted that subsequent pharmacological models have shown that using a two- or three-compartment PK model, or allowing for conversion to an active metabolite are more appropriate for some anti-malarials (see for example [12, 29]). The purpose of the PK model within OptiMal-PK is to inform the development of a drug candidate so the assumption of a one-compartment model is a reasonable and pragmatic first step.

It is important to note that the model makes a large number of other simplifications similar to previous models. For example, OptiMal-PK does not currently include bioavailability, dose dependency, active metabolites and intercessional/intersubjective variability which are known for many drugs. However, the simple nature of the interface will allow the user to probe the effects of these variables and assess their effect. For instance, the effect of bioavailability can be easily explored by reducing or increasing the drug dosage, or by altering the clearance values. Active metabolites can be modelled by adding an extra drug with parameters that match that of the metabolite and whose absorption rate is equal to the conversion rate to the metabolite.

For combination therapy simulations, a model of dominant killing was utilised, where the rate of kill is equal to that of the drug inferring the fastest kill rate at each time point as previously described in [12].

### A case study—Is OZ439 sufficiently effective for delivery as a one dose therapy and how it could it be improved?

OZ439 is a peroxide anti-malarial drug that has been designed to produce a single oral dose cure for malaria [30]. Unlike other peroxides, OZ439 showed excellent activity in *Plasmodium berghei*-infected mice producing a complete cure after a single 30 mg/kg dose administered orally. The clinical PK data for the drug were described in a later publication [31] and showed superior PK properties when compared to similar drugs within the same class, with an elimination half-life that exceeds 24 h due partially to a relatively slow clearance. However, the question remains of whether OZ439 could truly deliver a single dose cure (defined as the reduction of total number of parasites to less than one) when administered as a single therapy in clinical studies, although it is accepted that the drug will be eventually deployed as a combination. OptiMal-PK was utilised to predict the clinical performance of OZ439 in future human studies. A number of simulations were executed assuming different dosages and feeding states and then allowed the program to assess the minimum number of days needed to achieve total elimination of parasites in the subjects under each scenario.

The OptiMalPK model calculated minimum number of days needed for treatment using the PK and PD parameters that are presented in Table 1 (derived from [30–32]) In this example, different doses of OZ439 were compared in a capsule and oral dispersion forms on empty (lower exposure) or full (higher exposure) stomachs based on clinical data. The results demonstrate that a single monotherapy dose of 800 or 1200 mg (as a capsule, it is important to note that the disposition characteristics of OZ439 capsules differ significantly from a drug dispersion formulation, see below) would be insufficient for the successful elimination of parasites in all patients and would result in recrudescence within 9 days after the treatment. The model predicts that 1200 mg OZ439 capsule would need at least 2 days of treatment to completely cure a malaria infection, as defined by depletion of parasite total number to one.

**Table 1:** PK and PD parameters of OZ439 after oral delivery in fasted and fed patients as reported in the literature

Parameter	Estimated value			Reference
	Fasted (capsule)	Fed (oral dispersion)	Fasted (oral dispersion)	
Dose (mg)	800–1200	800	800	
Absorption rate (ka)	0.72	0.72	1.5	[31]
Elimination half-life (h)	27.9	31.7 (healthy) 58.0 (malaria)	38.8	[31] [32]
Clearance (L/h/kg)	2.43	0.48 (healthy) 0.58 (malaria)	1.41 N/A	[31] [32]
Parasite reduction rate (PRR) (/48 h)	3000	3000	3000	Assumed to be similar to artemisinin
IC_50_ (nM)	4.5	4.5	4.5	[30]
Molecular weight (Da)	469.6	469.6	469.6	[30]
Hill’s slope constant	4	4	4	

**Fig. 3:**
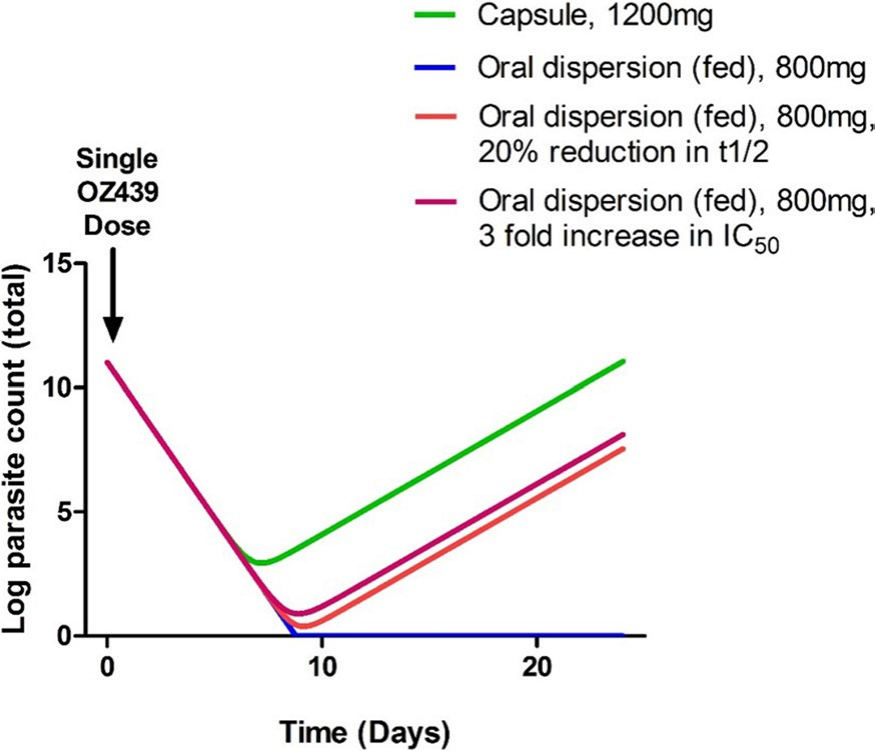
OptiMal-PK predictions of OZ439 clinical activity. Results of OptiMal-PK simulations on OZ439. Assuming a single dose of either 1200 mg capsule (fasted, *green line*), oral dispersion in fed subjects with (*blue*) or without (*red*) an assumption of 20 % reduction in t_1/2_ or a threefold increase in IC_50_ (*purple*)

However, the simulations show that a single 800 mg dose of the oral dispersion formulation in fed or fasted subjects would lead to a cure following a single administration; administering the drug in the capsule form, not the oral dispersion formulation, seems to fail a 1 day treatment regimen according to the model. Nevertheless, even for the superior oral dispersion formulation, if metabolism is slightly accelerated (decreasing half-life by 20 %) in a proportion of the target population, the single dose for the oral dispersion would be rendered insufficient in either fed or fasted subjects. These data suggest that the single dose regimen is very unforgiving which could have very serious consequences in field deployment in large patient populations if OZ439 were to be deployed alone. Additionally a modest two to threefold decrease in potency (increase in IC_50_ from x–y; i.e. emergence of moderate resistance) would also make the single dose insufficient for an absolute cure (Fig. 3). OptiMal-PK highlights the sensitivity of single dose OZ439 to emergence of low level parasite resistance and moderate population PK variations. Given the large variability of PK parameters shown in Table 1 (30–115 %) the simulations here show that a single dose of OZ439 dispersion would leave a significant proportion of subjects at risk of incomplete elimination of malaria parasites, treatment failures and parasite recrudescence and increased pressure for resistance development.

### Case study 2—effect of resistance on drug activity and mitigating strategies

**Fig. 4:**
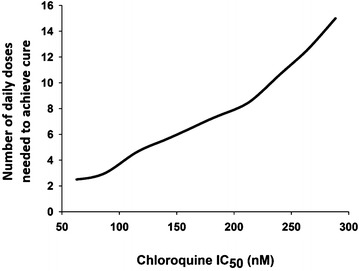
OptiMal-PK predictions of chloroquine resistance upon its activity. Effect of resistance on outcome of chloroquine (CQ) treatment: The graph show the increase in number of days needed to achieve a cure with CQ at increasing IC_50_ values from 62.7 up to 288.4. Increasing IC_50_ values indicate increased resistance to the drug

Chloroquine (CQ) was for many decades a primary chemotherapeutic agent in anti-malarial therapy. Resistance to the 4-aminoquinolines was initially reported in Southeast Asia and South America and subsequently spread to almost every malaria endemic region in the world [33]. CQ was used as an example on how drug resistance would affect the outcome of treatment. The original chloroquine parameters are all based on those previously reported as described earlier [11]. The number of days it takes to achieve a cure was investigated as drug resistance increases (IC_50_ was elevated in 40 % increments, Fig. 4). Using CQ sensitive IC_50_ values (e.g. ≤62.7 nM [34]), the model predicts successful outcome with a 3-day treatment, consistent with published clinical data [35], and consistent with WHO guidelines for CQ [36]. When parasite CQ sensitivity values are increased to resistant values e.g. generally considered ≥100 nM [37, 38], the model predicts a requirement of 4 or more days of treatment, depending on the fold increase of CQ C_50_ (Fig. 4), all PK parameters derived from [39] and PD parameters from [34]. These data are in agreement with clinical trials performed in CQ resistant regions in Afghanistan where 60 % of the population failed a 5-day treatment of CQ [40].

Parameters that could be altered to reverse the effect of increasing the IC_50_ of CQ were then investigated. Assuming a 4.6-fold increase of IC_50_ from 62.7 to 288.4 nM the drug will not be able to deliver a cure within 14 days, altering the dosing regimen from once daily dosing to twice daily brings the number of days needed to achieve a cure to five to 6 days according to the model, while artificially increasing the half-life by 40 % would reduce the predicted number of days needed to achieve a cure down to seven to 8 days. Increasing the PRR by tenfold from 1000 only improves the activity of CQ slightly according to the model. Although these changes are purely an academic exercise it highlights the key parameters that need to be considered in the discovery process where candidate selection could take into account these pharmacological features. Importantly, even if it were possible to achieve radical changes in the drug properties, such as increasing its PRR by tenfold or its half-life by 40 %, these changes are unable to mitigate resistance resulting from a 4.6-fold increase in the CQ IC_50_, emphasizing the need to avoid resistance development in the first case by appropriate use of forgiving dosage regimens.

### Case study 3—studying the potential of long-duration dihydroorotate dehydrogenase (DHODH) inhibitor (DSM265) for treatment of malaria

OptiMal-PK is used here to study the therapeutic potential of a drug, DMS265, with slow elimination kinetics and killing dynamics. Potency and clinical PK properties were used as previously reported [26] and shown in Table 2 (PRR value was estimated to be 17 based on data shown in [26]).

**Table 2:** PK and PD parameters of DSM265 as reported in the literature

Parameter	Estimated value
Dose (mg)	200–400 mg
Absorption rate (ka)	N/A (assumed to be 1)
Elimination half-life (h)	130 h
Clearance (L/h/kg)	0.003 (allometrically scaled from rodents)
Parasite reduction ratio (PRR) (/48 h)	17
IC_50_ (nM)	13
Molecular weight (Da)	402
Hill’s slope constant	~1 (extrapolated from graph)

Human PK values are reported from allometric scaling performed by [26]

The simulations show that using the parameters in Table 2, at least four doses would be needed to achieve complete elimination of parasites in the circulation of patients. However, if metabolism is slowed down by a factor of only 10–20 % (by increasing half-life from 130 to 170 h) the drug would be capable of achieving a cure in one to two doses. These data, therefore, suggest that in back-up programmes aimed at delivering second-generation DHODH inhibitors, a focus on improving the therapeutic half-life of the drug may be more rewarding than a focus on potency alone.

Like OZ439, albeit with a completely different mode of action and different PK/PD properties, DSM 265 seems to be on the cusp of delivering a single dose cure. As with OZ439, the laudable aim of achieving a single dose cure, needs to take into account inherent parasite and patient variability at the population level, as the models suggest that these drugs may end up being deployed in very unforgiving dosage regimens, setting them up for premature failure due to either inadequate clinical performance in some patients or the early development of parasite resistance, something that is readily achieved with DSM265 in laboratory settings under moderate drug pressure.

## Conclusions

OptiMal-PK is an open access, internet-based tool designed for the wider anti-malarial drug discovery community. It allows non-specialists to identify which pharmacological features of a compound are most important to the clinical performance of a new chemical entity or new drug combination. OptiMal-PK should therefore serve as an aid to critical decision-making during anti-malarial drug development programmes.

## Abbreviations

ADMET: absorption, disposition, metabolism, excretion and toxicity
C: concentration
CL: clearance
CQ: chloroquine
DHA: dihydroartemisinin
DMPK: disposition, metabolism and pharmacokinetics
IC: inhibitory concentration
MPC: minimum parasitic concentration
PD: pharmacodynamics
PK: pharmacokinetics
PMR: parasite multiplication ratio
PRR: parasite reduction ratio
MPC: minimum parasitic concentration
*k*_*a*_: absorption rate
*k*_*e*_: elimination rate
QSAR: quantitative structure–activity and relationships
T_1/2_: elimination half-life
TCP: target candidate profile
TPP: target product profile
V_max_: maximal kill rate
V_d_: volume of distribution
